# Insight Into the Potential Value of Gut Microbial Signatures for Prediction of Gestational Anemia

**DOI:** 10.3389/fcimb.2021.734561

**Published:** 2021-08-30

**Authors:** Hongcheng Wei, Siting Deng, Yufeng Qin, Xu Yang, Ting Chen, Xu Wang, Yankai Xia

**Affiliations:** ^1^State Key Laboratory of Reproductive Medicine, School of Public Health, Nanjing Medical University, Nanjing, China; ^2^Key Laboratory of Modern Toxicology of Ministry of Education, School of Public Health, Nanjing Medical University, Nanjing, China; ^3^Nanjing Maternity and Child Health Care Institute, Women’s Hospital of Nanjing Medical University, Nanjing Maternity and Child Health Care Hospital, Nanjing, China; ^4^Department of Endocrinology, Children’s Hospital of Nanjing Medical University, Nanjing, China

**Keywords:** 16S rRNA gene sequencing, gut microbiota, co-abundance group, prediction, the risk of developing gestational anemia

## Abstract

The gut microbiota alternations are associated with gestational anemia (GA); however, limited predictive value for the subsequent incidence of anemia in normal gestational women has been obtained. We sought to rigorously characterise gut dysbiosis in subjects with GA and explored the potential predictive value of novel microbial signatures for the risk of developing GA. A prospective cohort of subjects with GA (n = 156) and healthy control (n = 402), all of whom were free of GA in the second trimester, by 16S rRNA gene sequencing was conducted. Microbial signatures altered dramatically in GA compared with healthy control in the second trimester. *Megamonas*, *Veillonella*, and *Haemophilus* were confirmed to show differential abundances in GA after adjusting for covariates. On the contrary, *Lachnospiraceae* and *Blautia* were enriched in control. Microbial co-abundance group (CAG) network was constructed. Prospectively, CAG network relatively accurately predicted upcoming GA in normal pregnant women with an AUC of 0.7738 (95%CI: 0.7171, 0.8306) and the performance was further validated in Validation set (0.8223, 95%CI: 0.7573, 0.8874). Overall, our study demonstrated that alterations in the gut microbial community were associated with anemia in pregnancy and microbial signatures could accurately predict the subsequent incidence of anemia in normal pregnant women. Our findings provided new insights into understanding the role of gut microbiota in GA, identifying high-risk individuals, and modulating gut microbiota as a therapeutic target, thus improving quality of life and well-being of women and children.

**Graphical Abstract:**
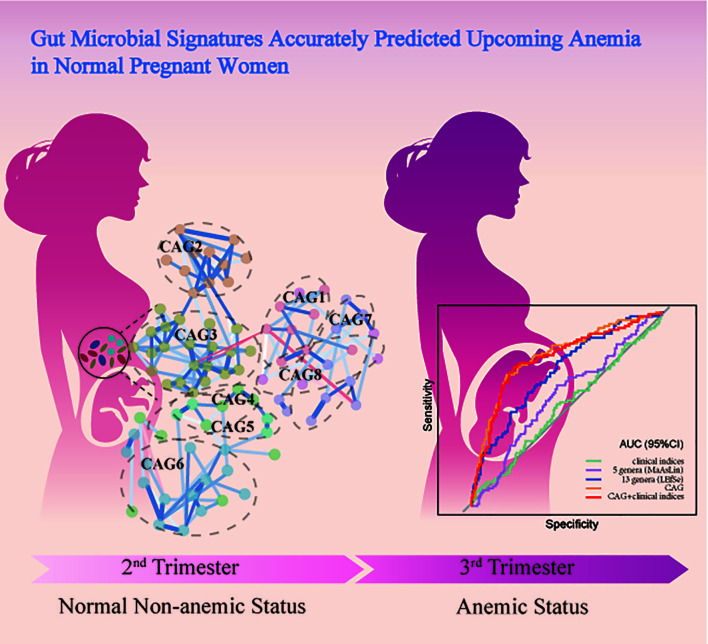


## Introduction

Anemia is a serious public health problem affecting people all over the world, and is one of the most frequent complications involved in pregnancy, imposing a tremendous toll on well-being of approximately 40% of pregnant women in China (Li et al., 2018). Gestational anemia, according to the World Health Organization (WHO), is defined as a hemoglobin concentration (Hb) < 110 g L^-1^. Gestational anemia mostly occurred in the second and third trimester (Zhao et al., 2018). Multiple factors account for gestational anemia, nutritional iron deficiency anemia (IDA) (approximately 75%) and folate megaloblastic deficiency anemia being the commonest (Sifakis and Pharmakides, 2000; Goonewardene et al., 2012).

Anemia in pregnancy has adverse effects on maternal and neonatal health. Weakness, fatigue, being vulnerable to infection, reduced work capacity, and productivity are typical symptoms during pregnancy (Prema et al., 1982; Hunt, 2002). Current evidence suggests severe gestational anemia could be associated with an increased risk of preterm birth, low birth weight, and even neonatal and maternal mortality (Khan et al., 2006; Rahman et al., 2016; Figueiredo et al., 2018; Guignard et al., 2021). Furthermore, it has been reported that babies born to anemic mothers are prone to exhibit future poor cognitive performance and delayed mental and motor development in adolescence and adulthood (Anker et al., 2009; Camaschella, 2015).

The current clinical method is generally applied to cross-sectional diagnosis rather than prediction in the long term. Although Hb concentration serves as a golden standard for the diagnosis of anemia in pregnancy, it has limitations in precise prediction of potential impending anemia in normal pregnant women. Additionally, it remains controversial to use Hb concentration to distinguish true or absolute anemia from relative anemia, ascribed to a normal physiologic increase of plasma volume (Sifakis and Pharmakides, 2000). Thus, an accurate determination of gestational anemia is essential and of enormous clinical importance for prevention and management of anemia in pregnancy.

The human gut hosts an immense number of resident microorganisms, collectively termed as the microbiota (Structure, Function and Diversity of the Healthy Human Microbiome, 2012; Bäckhed et al., 2004). There has been accumulating evidence that the gut microbiota is implicated in enteric, metabolic, and psychiatric diseases (Imhann et al., 2018; Vich Vila et al., 2018; Thomas et al., 2019; Wirbel et al., 2019). Previous studies have documented that gut microbial dysfunction (e.g., deficiency in *lactobacillus*) is related to IDA and gut microbiota could promote hematopoiesis, underlying the close relationship between gut microbiota and anemia (Balamurugan et al., 2010; Khosravi et al., 2014). During pregnancy, host profoundly remodeling of the gut microbiota could cause symptoms of metabolic syndrome, which might be related to transportation and storage of host Fe, and ultimately lead to the occurrence of gestational anemia (Koren et al., 2012). Microbial signatures have been elucidated to function as novel biomarkers to discriminate patients suffering from illness and healthy individuals (Liu et al., 2019; Wei et al., 2020). To date, an anemia classifier in pregnancy has been constructed (Long et al., 2021); however, no efforts have been made to predict the subsequent incidence of anemia in normal gestational women.

To reach a better understanding of how gestational anemia is developing and regulated, herein, we conducted a prospective study to rigorously evaluate dynamic landscape of gut microbiota varying from healthy status to identified anemic status in pregnant women. Leveraging the discriminative microbial signatures in the early stage of pregnancy when anemia did not occur yet, we prospectively built an accurate prediction model for the subsequent incidence of gestational anemia. Our findings help identify pregnant women with high risk of anemia in general population and ultimately improve quality of life and well-being of women and children.

## Materials and Methods

### Study Design and Participants

This study used data from the Mother and Child Microbiome Cohort (MCMC) Study, a prospective birth cohort study initiated and maintained in Nanjing Maternity and Child Health Care Hospital. The recruitment of eligible study pregnant women was from 2017 to 2018 (n = 1527), when they were all in the second trimester. This cohort aimed to explore the relationship between gut microbiota and maternal and children’s health.

Among 1527 eligible women who were enrolled, 781 subjects have provided fecal samples both in the second and third trimester. Then, those with assisted conception (n = 19) or twin pregnancy (n = 8) were excluded. Additionally, women with pregnant complications containing diabetes mellitus and pathological anemia (e.g., aplastic anemia and hemolytic anemia) were excluded (n = 142). Women with diagnosed anemia in the second trimester were excluded (n = 54). In the final analysis, 558 participants were included (**Supplementary Figure 1**).

Though Hb < 110 g L^-1^ has been the accepted criterion for the diagnosis of gestational anemia, we reduced the diagnostic level to Hb < 100 g L^-1^ considering the increasing plasma volume during pregnancy, to reduce bias (Lund and Donovan, 1967). All subjects accepted treatment after they were diagnosed with anemia in our cohort.

### Ethics

We obtained signed informed consent from all participants. The study was approved by the Ethics Committee of Nanjing Medical University [FWA00001501 No. (2017) 003].

### Sample Collection and Sequencing Data

All samples were collected prior to anemia treatment. Fecal samples were obtained from each participant for measuring gut microbiota during the second (at 24 weeks of pregnancy) and third trimester (at 32 weeks of pregnancy), respectively, and were stored at -80°C until DNA extraction. Serum samples were obtained during the second trimester and were frozen in -20°C freezers. Serum ferritin levels were measured by electrochemiluminescence immunoassay on the immunoassay analyzer (Beckman Coulter Inc., Fullerton CA, USA) with the same batch of reagents.

16S rRNA sequencing was performed using primers (338F: 5’-ACTCCTACGGGAGGCAGCAG-3’ and 806R: 5’-GGACTACHVGGGTWTCTAAT-3’). Each sample genomic DNA was extracted using QIAamp Fast DNA Stool Mini Kit (Qiagen, Germany). From extracted DNA, we sequenced the hypervariable region 16S rRNA gene V3 to V4 regions by HiSeq2500 PE250 platform in Meiji Bioinformatics Technology Co. Ltd (Nanjing, China). Blinded positive quality control (QC) specimens were used across all sequencing batches for quality control.

The 16S rRNA sequencing data were analyzed using Quantitative Insights Into Microbial Ecology (QIIME2 V.2020.6). The dada2 plugin was used to denoise sequences, and this quality control process will additionally filter any phiX reads (commonly present in marker gene Illumina sequence data) that were identified in the sequencing data, and will filter chimeric sequences. Amplicon sequence variants (ASVs) were obtained at 100% sequence homology; the taxonomy was assigned against the Silva database (Silva 138 release). To minimize the effect of spurious sequences, one case with too low sequence number was excluded. Representative sequences for each ASV were built into a phylogenetic tree with FastTree plugin. Alpha and beta diversity analyzes were conducted at a rarefied sampling depth of 31291.

### Statistical Analysis

To compare maternal anemia information by characteristics, *t*-test for continuous variables and *χ²* test for categorical variables were used. Multivariate linear regression model was applied to multiple-factor analysis. Model 1 was crude model; Model 2 was adjusted for maternal age, pre pregnant body mass index (BMI), parity, gravidity, family income, maternal education level, passive smoking, antibiotic use during pregnancy, folic acid and iron supplement use during pregnancy, alcohol and caffeine use pre pregnancy, and alcohol and caffeine use during pregnancy.

Wilcoxon rank-sum test was used to compare α-diversity, and permutational multivariate analysis of variance (PERMANOVA) using 9999 permutations was used to test for statistical significance between two groups. Given a false discovery rate (FDR) of 5%, linear discriminant analysis effect size (LEfSe) was used to identify bacteria differentially abundant between anemic women and normal women (Segata et al., 2011). To further validate the results, after adjusting for maternal age, pre pregnant BMI, parity, gravidity, family income, maternal education level, passive smoking, antibiotic use during pregnancy, folic acid and iron supplement use during pregnancy, alcohol and caffeine use pre pregnancy, and alcohol and caffeine use during pregnancy, multivariate association with linear models algorithm (MaAsLin) analysis was performed (Morgan et al., 2012; Wei et al., 2020).

The top 99 most abundant genera were used to construct co-abundance group (CAG) network. Kendall correlation was calculated by the function “cor”. CAG was defined with a Spearman correlation distance metric using the “Made4” R package. The appropriate number of clustering was selected based on significance testing among each group of the original Kendall correlation matrix using “adonis” function in “Vegan” R package. The sum of relative abundance of ASVs which belonged to the same CAG was calculated to represent the abundance of this CAG (Zhang et al., 2021). Subsequently, we used “qgraph” R package to construct the regularized partial correlation network based on least absolute shrinkage and selection operator (lasso) (Epskamp and Fried, 2018; Liang et al., 2020).

Spearman correlation was used to investigate relationships among continuous variables, and point biserial correlation was used to examine relationships between binary variable and continuous variables. Finally, we constructed receiving operational curve (ROC) and calculated area under curve (AUC) to assess the predictive performance of the model with the “pROC” R package.

## Results

### Characteristics of the Study Population

Totally, 156 (27.96%) women diagnosed with gestational anemia (GA) and 402 healthy control in the third trimester, all of whom were non-anemic in the second trimester, constituted the study population for final analysis. Detailed demographic features of the cohort were summarized in **Table 1**.

**Table 1:** Characteristics of the study cohort.

Characteristics	Healthy control(n = 402)	GA*(n = 156)	*P*
Age (years)	28.89 ± 3.19	29.39 ± 3.30	0.76
Pre pregnant BMI(kg/m^2^)	22.44 ± 5.91	21.85 ± 4.85	0.23
Fetal gender			
Boy	204 (50.75)	79 (50.64)	0.98
Girl	198 (49.25)	77 (49.36)	
Fetal BMI(kg/m^2^)	13.41 ± 1.26	13.79 ± 1.19	0.71
Gravidity			
1	238 (59.20)	90 (57.69)	0.75
≥2	164 (40.80)	66 (42.31)	
Parity			
0	303 (75.37)	122 (78.21)	0.48
≥1	99 (24.63)	34 (21.79)	
Family income (Chinese Yuan/year)			
<100,000	27 (6.72)	6 (3.85)	0.21
100,000–200,000	164 (40.80)	53 (33.97)	
>200,000	30 (7.46)	16 (10.26)	
Maternal education level			
College and below	86 (21.39)	36 (23.08)	0.81
University	156 (38.81)	57 (36.54)	
Graduate school and above	47 (11.69)	20 (12.82)	
Passive smoking			
Never	123 (30.60)	54 (34.62)	0.06
Seldom	159 (39.55)	64 (41.03)	
Always	32 (7.96)	4 (2.56)	
Antibiotic use before in the early stage			
No	167 (41.54)	55 (35.26)	0.10
Yes	205 (51.00)	94 (60.26)	
Folic acid and iron supplement use in the early stage			
No	28 (6.97)	4 (2.56)	0.09
Yes	374 (93.03)	152 (97.44)	
Alcohol use pre pregnancy			
No	246 (61.19)	94 (60.26)	0.97
Yes	18 (4.48)	7 (4.49)	
Caffeine use pre pregnancy			
No	158 (39.30)	64 (41.03)	0.42
Yes	125 (31.09)	42 (26.92)	
Alcohol use during pregnancy			
No	270 (67.16)	99 (63.46)	
Yes	0 (0.00)	0 (0.00)	
Caffeine use during pregnancy			
No	238 (59.20)	91 (58.33)	0.23
Yes	44 (10.95)	11 (7.05)	

Data presented by mean ± SD or n (%).

*GA (gestational anemia), based on the diagnosis of objects in the third trimester.

### Fecal Microbiota Altered Dramatically During Pregnancy

**Figure 1:**
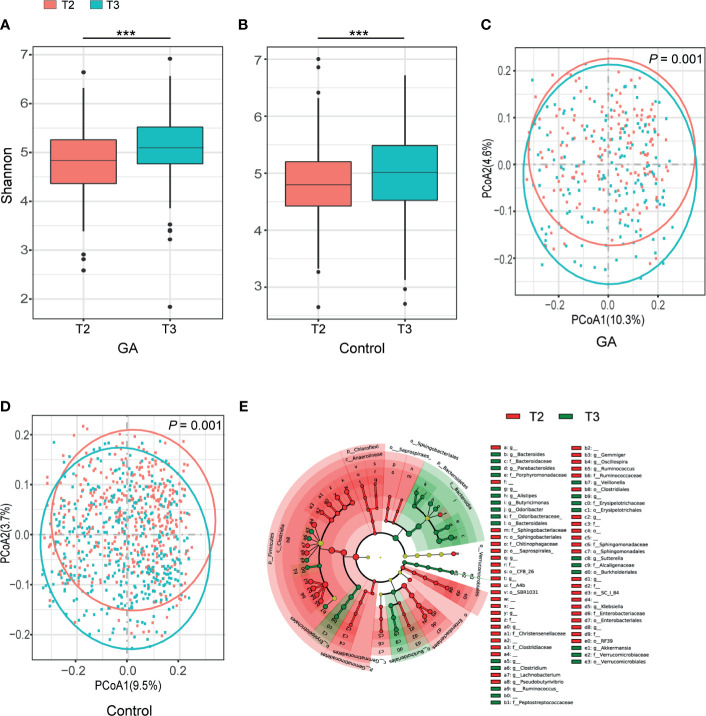
Comparisons of alpha-diversity, beta-diversity, and variations of gut microbiota composition between T2 and T3. **(A)** Box plots based on Shannon diversity index in GA group. **(B)** Box plots based on Shannon diversity index in healthy control group. **(C)** PCoA based on unweighted UniFrac matrix of GA group. **(D)** PCoA based on unweighted UniFrac matrix of healthy control group. **(E)** The cladogram showed differently enriched taxa in the second trimester and the third trimester (FDR < 0.05). FDR, false discovery rate; PCoA, principal coordinate analysis; GA, gestational anemia; T2, the second trimester; T3, the third trimester; ***P < 0.001.

Shannon index indicated progression of pregnancy from the second trimester (T2) to the third trimester (T3) was accompanied by an increment in α-diversity (GA: *P* < 0.001, Control: *P* < 0.001, **Figures 1A, B**) and the observed ASVs suggested the same trend (**Supplementary Table 1**). Principal coordinate analysis (PCoA) based on unweighted UniFrac distance was conducted to elaborate the overall structure of microbial composition. PERMANOVA manifested significant differences in structure and composition of the microbiota between T2 and T3 (GA: *P* = 0.001, Control: *P* = 0.001, **Figures 1C, D**). Such significant difference was also observed based on weighted UniFrac distance (**Supplementary Table 1**). LEfSe analysis revealed that remarkable differences were observed between T2 and T3 in pooled group (LDA > 2, FDR < 0.05, **Figure 1E** and **Supplementary Table 2**).

### Changes in the Gut Microbiota Between GA and Healthy Control

**Figure 2:**
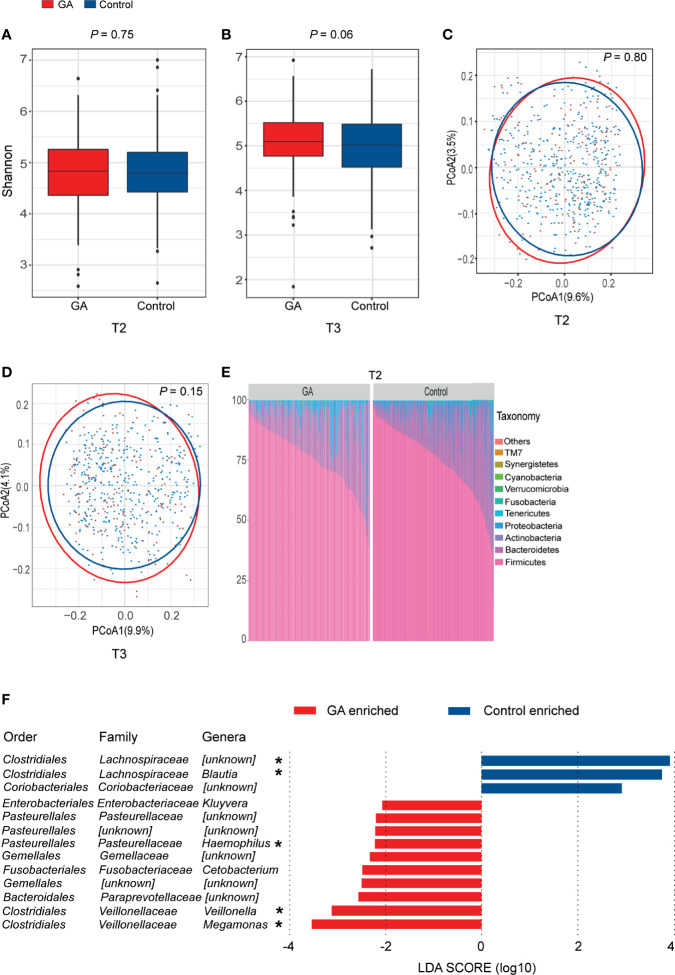
Comparisons of alpha-diversity, beta-diversity, and variations of gut microbiota composition between GA and healthy control. **(A)** Box plots based on Shannon diversity index in the second trimester. **(B)** Box plots based on Shannon diversity index in the third trimester. **(C)** PCoA based on unweighted UniFrac matrix of T2 group. **(D)** PCoA based on unweighted UniFrac matrix of T3 group. **(E)** Relative proportions of bacterial phyla in GA and healthy control in the second trimester. **(F)** Histogram of the LDA scores computed for differentially abundant taxa between GA and healthy control in the second trimester (FDR < 0.05). FDR, false discovery rate; PCoA, principal coordinate analysis; GA, gestational anemia; T2, the second trimester; T3, the third trimester; LDA, linear discriminant analysis; *Genera remained significantly associated with GA after adjusting for covariates using multivariate association with linear models algorithm (MaAsLin). *P < 0.05.

Both Shannon index (T2: *P* = 0.75, T3: *P* = 0.06, **Figures 2A, B**) and the observed ASVs (**Supplementary Table 1**) in different trimesters showed that there was no difference in richness and diversity of the gut microbiota between GA and healthy control. No significant difference was observed between GA and healthy control based on unweighted or weighted UniFrac distance in both T2 and T3 (T2: *P* = 0.80, T3: *P* = 0.15, **Figures 2C, D** and **Supplementary Table 1**).

In both T2 and T3, overall microbiota composition of maternal fecal microbiota at phylum level showed that the maternal fecal microbiota was dominated by *Firmicutes* and *Bacteroidetes*, implying there was no significant change between GA and healthy control at the phylum level (**Figure 2E** and **Supplementary Figure 2**). In the second trimester, 10 bacterial taxa, including *Megamonas*, *Veillonella*, *Paraprevotellaceae*, *Gemellales*, *Cetobacterium*, *Gemellaceae*, *Haemophilus*, *Pasteurellales*, *Pasteurellaceae*, and *Kluyvera* were observed enriched in GA versus control (LDA > 2, FDR < 0.05, **Supplementary Table 3**). *Megamonas*, *Veillonella*, and *Haemophilus* were confirmed to show differential abundances between GA and healthy control after validated by MaAsLin analysis. On the contrary, *Lachnospiraceae* and *Blautia* were enriched in control (**Figure 2F**). Detailed information was shown in **Supplementary Table 4**. In the third trimester, after adjusting for covariates, increased abundance in *Veillonella* and decreased abundance in *Lachnospiraceae* and *Blautia* were observed in GA (**Supplementary Figure 2**).

### Microbial Co-abundance Group Network

**Figure 3:**
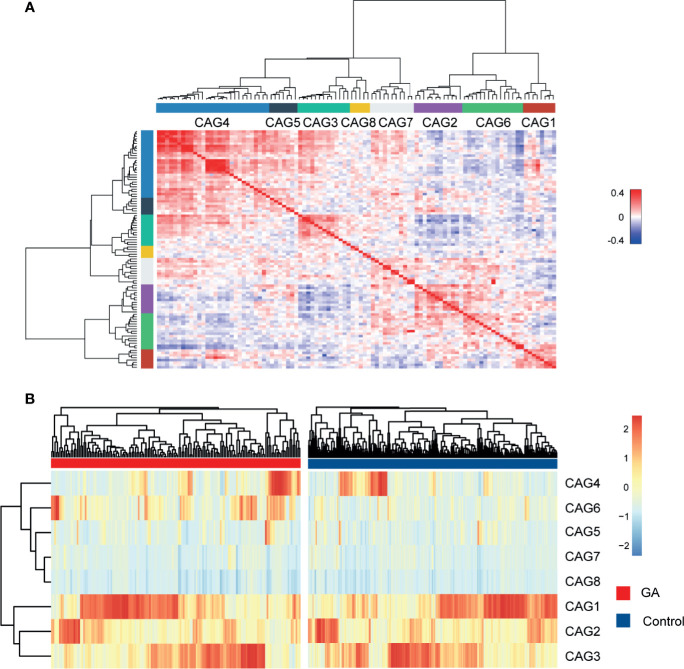
Heatmap of microbial co-abundance group. **(A)** Kendall correlations coefficients between the top 99 most abundant genera in the second trimester were calculated, and eight CAGs were clustered based on Kendall correlation matrix. **(B)** CAG differently enriched in GA and healthy control in the second trimester. GA, gestational anemia; CAG, co-abundance group.

**Figure 4:**
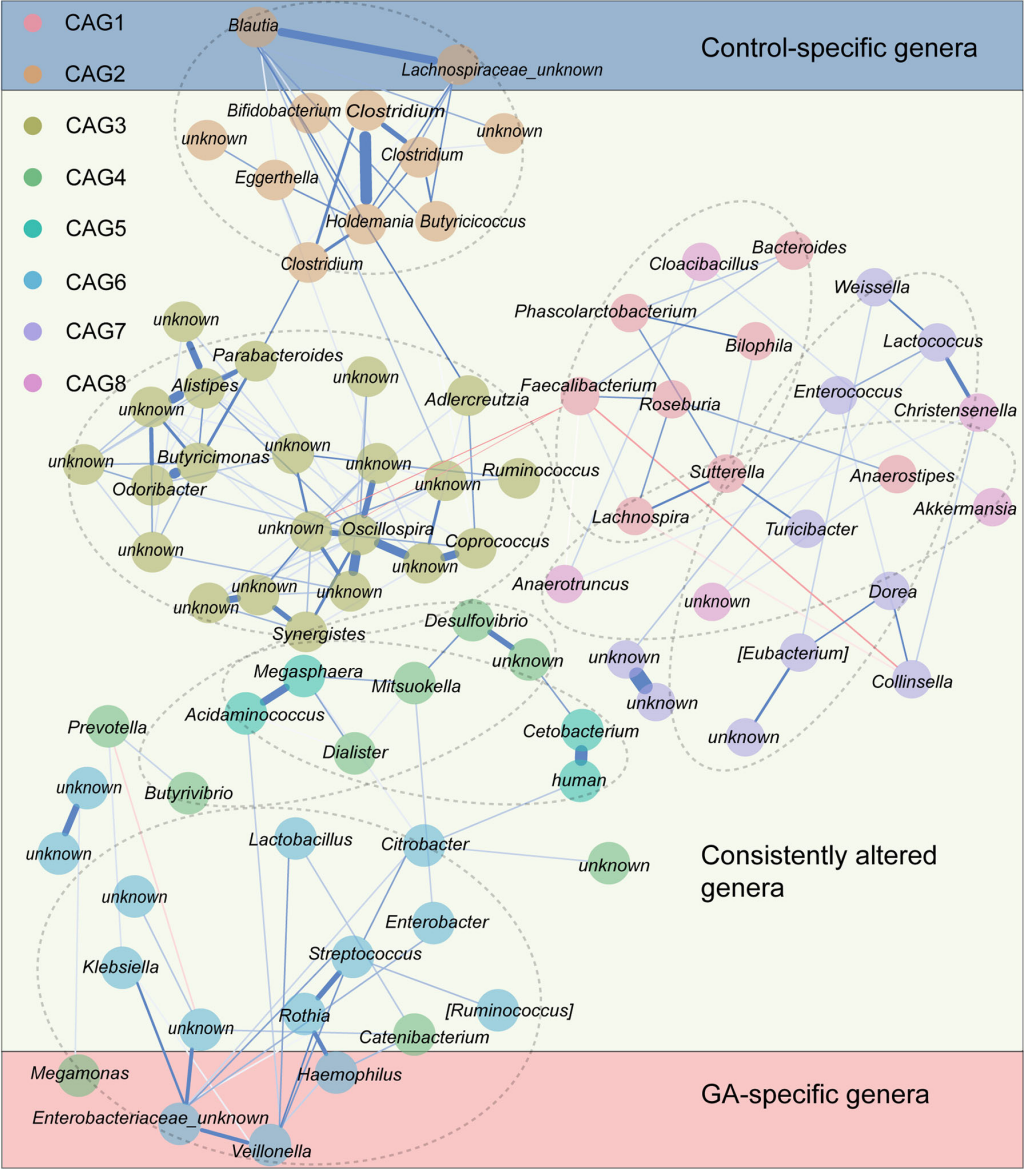
Co-abundance group network reflecting microbial changes in GA and healthy control. Regularized partial correlation network of top altered taxa in GA in the second trimester. Each node represented a taxon, and each edge represented the strength of partial correlation between two taxa. Edge weights represented the partial correlation coefficients. Blue edge represented positive correlation, and red edge represented negative correlation. GA, gestational anemia.

Since bacteria work as functional groups (Zhang et al., 2015), in the second trimester, the top 99 most abundant genera were clustered into 8 CAGs according to their co-abundance correlations (**Figures 3A, B**), and the regularized partial correlation network based on lasso regression was constructed (**Figure 4**). CAG1, CAG2, CAG3, and CAG4, accounting for 86.36% of ASVs, were consistently abundant both in GA and healthy control. Intriguingly, the significantly enriched taxa in GA belonged to CAG6 and the significantly enriched taxa in healthy control were specific to CAG2. These findings suggested a highly coordinated microbial regulatory network might underlie the occurrence of gestational anemia. Detailed information on CAG clusters was shown in **Supplementary Table 5**.

### Interrelationship Between Gut Microbiota Composition, Clinical Indices, and GA

**Figure 5:**
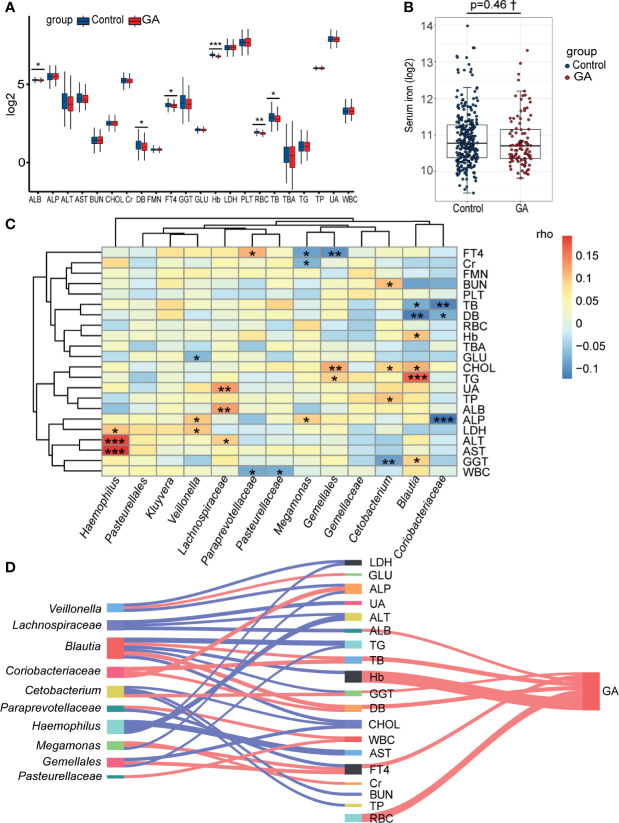
Correlations among the gut microbiota, clinical indices, and GA. **(A)** The box plot showed that the clinical indices significantly changed between two groups. **(B)** The difference of levels of serum iron in the second trimester between the two groups. **(C)** The heatmap of the partial Spearman correlations between gut microbiota and clinical indices in the second trimester (FDR < 0.05). **(D)** Relationships among gut microbiota composition, clinical indices, and GA (only significant correlations were presented, FDR < 0.05). The width of lines represented the partial correlation coefficients. Red line represented negative correlation and blue line represented positive correlation. GA, gestational anemia; FDR, false discovery rate; *P < 0.05, **P < 0.01, ***P < 0.001; †Adjusted for potential covariates.

The abundance comparison adjusted for potential confounders showed decreased albumin (ALB), direct bilirubin (DB), free thyroxine (FT4), Hb, red blood cell (RBC), and total bilirubin (TB) were significantly associated with GA in the second trimester (**Figure 5A** and **Supplementary Table 6**). Intriguingly, no significant association was observed between GA and serum iron after adjusting for covariates (**Figure 5B** and **Supplementary Table 7**). Considering an FDR of 5%, the partial Spearman correlation delineated that 13 differentially abundant bacterial taxa were significantly correlated with clinical indices (**Figure 5C** and **Supplementary Tables 8**, **9**). As the Sankey plots demonstrated, in the second trimester, *Megamonas* was negatively correlated with FT4 and further negatively correlated with GA, and *Blautia* was positively correlated with Hb and further negatively correlated with GA, indicating that gut microbiota could be involved in occurrence of anemia by interacting with clinical indices (**Figure 5D**).

### Potential Predictive Value of Gut Microbial Signatures for GA

**Figure 6:**
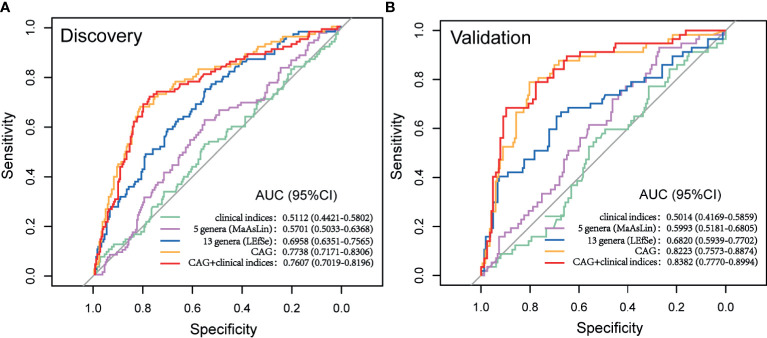
Prediction model of GA based on microbial signatures. **(A)** Discovery set (GA: 91; control: 244). **(B)** Validation set (GA: 65, control: 158). GA, gestational anemia; AUC, area under curve.

The cohort was further randomly divided into a Discovery set (GA: 91; Control: 244) and a Validation set (GA: 65; Control: 158). As shown in **Figure 6**, clinical indices alone had a poor performance in predicting upcoming anemia (Discovery AUC: 0.5112, 95%CI: 0.4421, 0.5802; Validation AUC: 0.5014, 95%CI: 0.4169, 0.5859). Further on, the potential value of gut microbiota acting as predictor was assessed. Using five genera adjusted by MaAsLin generated an AUC of 0.5701 (95%CI: 0.5033, 0.6368) in Discovery set and 0.5993 (95%CI: 0.5181, 0.6805) in Validation set. Using 13 genera based on LEfSe yielded an AUC of 0.6958 (95%CI: 0.6351, 0.7565) in Discovery set and 0.6820 (95%CI: 0.5939, 0.7702) in Validation set. Of note, CAG accurately predicted an upcoming GA with an AUC of 0.7738 (95%CI: 0.7171, 0.8306) and the classifying ability was further validated in Validation set (0.8223, 95%CI: 0.7573, 0.8874). No significant improved predictive performance was observed when including the combination of CAG and clinical indices into the prediction model (Discovery AUC: 0.7607, 95%CI: 0.7019, 0.8196; Validation AUC: 0.8382, 95%CI: 0.7770, 0.8994).

## Discussion

In the current study, we delineated that GA microbial dysbiosis was characterized by several bacterial genera and structured CAG. A cross-sectional anemia classifier in the first trimester and second trimester has been constructed (Long et al., 2021); however, limited efforts have been made to prospectively predict future anemic women. The greatest advantage was that for the first time, a prediction model for upcoming anemia in normal pregnant women with relatively high discriminatory power was established based on novel gut microbial signatures.

It was challenging to determine the etiology of anemia and confirm IDA in pregnancy. Apart from Hb, more laboratory examinations (e.g., serum iron, transferrin receptor, transferrin saturation, and bone marrow iron) should be taken into consideration. The most common true anemia in pregnancy was IDA. In addition, there were no other types of pathological anemia (e.g., aplastic anemia and hemolytic anemia) remaining in our study. Thus, anemic women were basically postulated to suffer from IDA.

Clinical indices serve as generally accepted diagnostic criteria for GA cross-sectionally; however, they were confirmed to have very limited predictive value for potential impending anemia in normal pregnant women according to our study. Gut microbial signatures exhibited impressive performance in the prediction model. Bacteria were significantly differently abundant in GA and healthy control in the second trimester. Of note, 93.03% of healthy control and 97.44% of anemic women took folic acid and iron supplement from conception to the second trimester, implying the sufficient iron storage. From the perspective of potential mechanism, the altered gut microbiota in the early stage was conjectured to be subsequently associated with the altered health condition (i.e., from iron sufficiency to iron deficiency or malnutrition) and further accounted for the upcoming anemia.

*Megamonas*, *Veillonella*, and *Haemophilus* were enriched in GA. *Megamonas* could act as a beneficial bacterium, and it has been reported that compared to healthy microbiota, canine anal furunculosis (CAF) microbiota showed a decreased abundance of *Megamonas* (Maldonado-Contreras et al., 2020). Nevertheless, another study illustrated infant vitamin D supplementation was associated with a lower abundance of *Megamonas* in gut microbiota, implying the potential competitive relationship between vitamin D and *Megamonas* (Drall et al., 2020). *Veillonella* species, documented as the Fe (III)-reducing genera, were capable of supplying Fe (II) to combine with oxygen in Hb (Jin et al., 2019). We hypothesized there might be a negative feedback regulation, host generating more *Veillonella* species when detecting less Hb combined with Fe (II). *Haemophilus* is a genus of Gram-negative, containing several markedly pathogenic bacteria, such as *Haemophilus influenzae* causing septicemia. Indigenous bacteria might inhibit host Fe transport and storage *via* producing metabolites that suppress hypoxia-inducible factor 2α (HIF-2α), assumed as a master transcription factor of intestinal Fe absorption and increasing the Fe-storage protein ferritin (Das et al., 2020). Decreased incidence of *Blautia* has been detected in the gut microbiota of obese children and *Blautia* genera might help to reduce inflammation causally linked to obesity-related complications (Benítez-Páez et al., 2020).

Microbial network has been an increasingly popular tool to explore microbial community structure (Röttjers and Faust, 2018). Ecologically, gut microbiota exists in functional groups named “guilds” rather than isolation and thrives in communities with large numbers and develops close interactions, which are critical evolutionary pressures for natural selection in microbial evolution (Faust and Raes, 2012; Ma et al., 2020). We sought to reduce the dimensionality of microbial datasets to identify GA more effectively based on CAG, and interestingly, the prediction model exhibited a much higher discriminatory power.

It is noticeable that normal physiologic changes in pregnancy lead to a relative or absolute reduction in Hb concentration. However, it is still an open question that this “anemia” is physiologic or pathologic. Given gut microbial signatures were free from influence of hydremia, a better diagnostic or predictive performance using gut microbial signatures could be achieved when it comes to either true anemia or physiologic anemia.

There were some advantages of our study. Firstly, samples were collected prior to treatment initiation in a large and well-characterized cohort. Secondly, antibiotics use was controlled in analysis. On the other hand, most of pregnant women enrolled in the study claimed antibiotics were used very early in pregnancy at a low dose and once they were discovered to be pregnant, antibiotics use was avoided as much as possible, which meant a possible lesser impact of antibiotics on gut microbiota. Thirdly, we constituted the first exploration to prospectively predict the risk of anemia in healthy subjects based on gut microbiota. Lastly, a much more accurate prediction model was built based on CAG network.

There were several limitations. Firstly, as we have discussed above, diagnostic evidence of IDA was not sufficient enough. Secondly, we did not construct a GA classifier using gut microbiota in the third trimester since that Hb was supposed to be a better alternative, quicker and costing lower. There was no information on vitamin D levels or supplementation in women, which was supposed to be a factor associated to gut dysbiosis and consequently GA. In addition, lack of metagenomics sequencing limited data interpretation from the angle of species level and bacterial function. Finally, there was not an independent cohort to verify the prediction model; our study merely provided evidence of association rather than causality and further studies are supposed to be conducted to validate the association.

## Conclusions

Our results showed that alterations in the gut microbial community were associated with anemia in pregnancy. Moreover, microbial signatures relatively accurately predicted the subsequent incidence of anemia in normal pregnant women. Our findings could provide new insights into understanding the role of gut microbiota in GA, identifying high-risk individuals, and modulating gut microbiota as a therapeutic target, thus improving quality of life and well-being of women and children.

## Data Availability Statement

The original contributions presented in the study are included in the article/**Supplementary Material**. Further inquiries can be directed to the corresponding authors.

## Ethics Statement

The studies involving human participants were reviewed and approved by the Ethics Committee of Nanjing Medical University [FWA00001501 No. (2017) 003]. The patients/participants provided their written informed consent to participate in this study.

## Author Contributions

Conceptualization: XW and YX. Formal analysis: HW and SD. Methodology: HW and YQ. Writing original draft: HW and SD. Verifying the underlying data: YQ and XY. Review and editing: YQ, XY, TC, XW, and YX. All authors contributed to the article and approved the submitted version.

## Funding

This work was supported by China-U.S. Program for Biomedical Collaborative Research (NSFC-NIH) (81961128022), the fifth phase of “333 High-level Talent Training Project” of the Jiangsu Province (BRA2020070), Postgraduate Research & Practice Innovation Program of Jiangsu Province (JX10313741), and the Priority Academic Program Development of Jiangsu Higher Education Institutions (PAPD).

## Conflict of Interest

The authors declare that the research was conducted in the absence of any commercial or financial relationships that could be construed as a potential conflict of interest.

## Publisher’s Note

All claims expressed in this article are solely those of the authors and do not necessarily represent those of their affiliated organizations, or those of the publisher, the editors and the reviewers. Any product that may be evaluated in this article, or claim that may be made by its manufacturer, is not guaranteed or endorsed by the publisher.

## Supplementary Material

Supplementary Figure 1 Flow chart of the inclusion of subjects.

Supplementary Figure 2 **(A)** Relative proportions of bacterial phyla in GA and healthy control in the third trimester. **(B)** Histogram of the LDA scores computed for differentially abundant taxa between GA and healthy control in the third trimester. GA, gestational anemia; LDA, linear discriminant analysis; *Genera remained significantly associated with GA after adjusting for covariates using multivariate association with linear models algorithm (MaAsLin).
