# Supplementary figures and images for: Insight Into the Potential Value of Gut Microbial Signatures for Prediction of Gestational Anemia
Source: Front Cell Infect Microbiol. 2021 Aug 30;11:734561. doi: 10.3389/fcimb.2021.734561 (PMC8437374; doi:10.3389/fcimb.2021.734561)

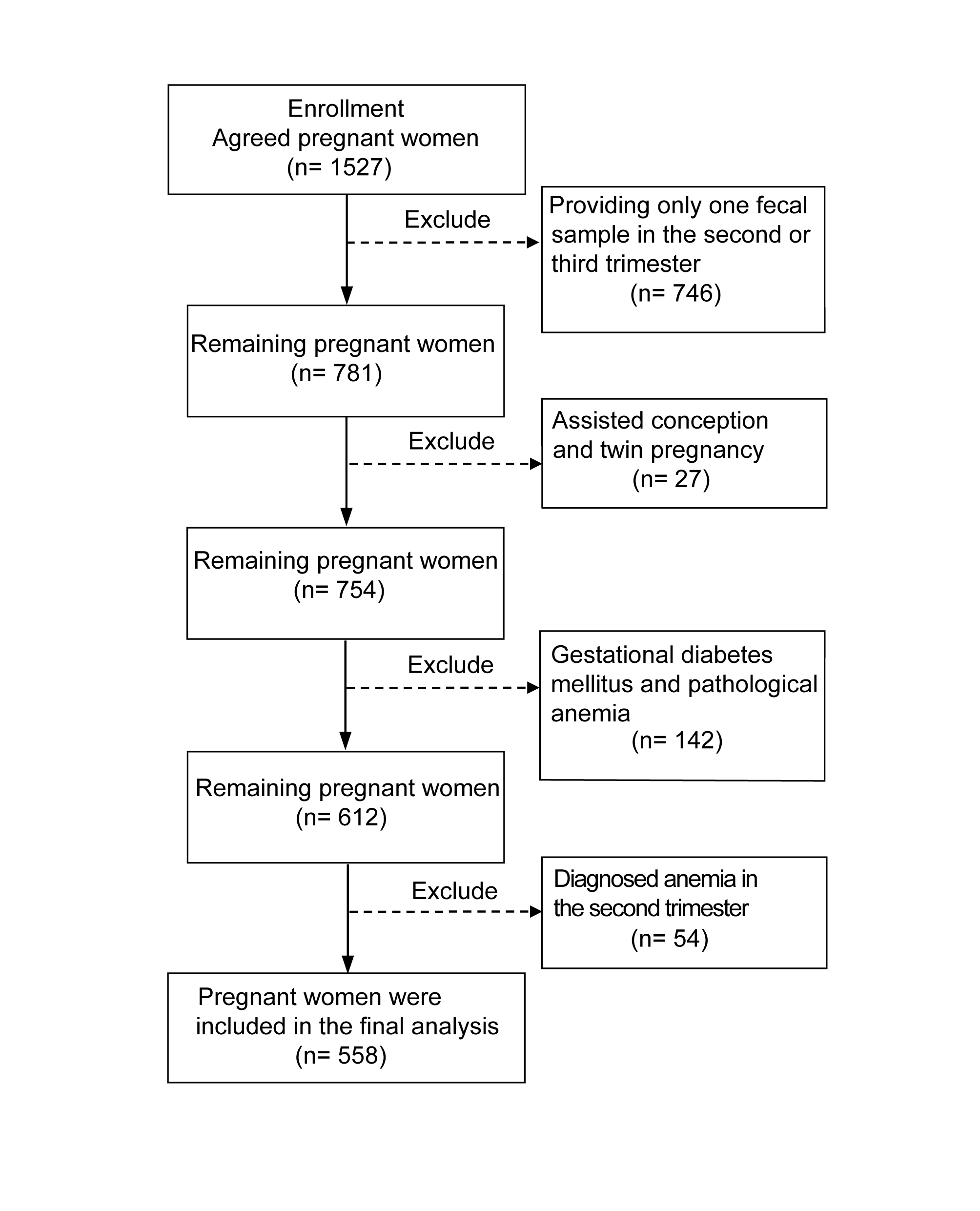

Supplement: Supplementary Figure 1 — Flow chart of the inclusion of subjects. [file Image_1.tif]

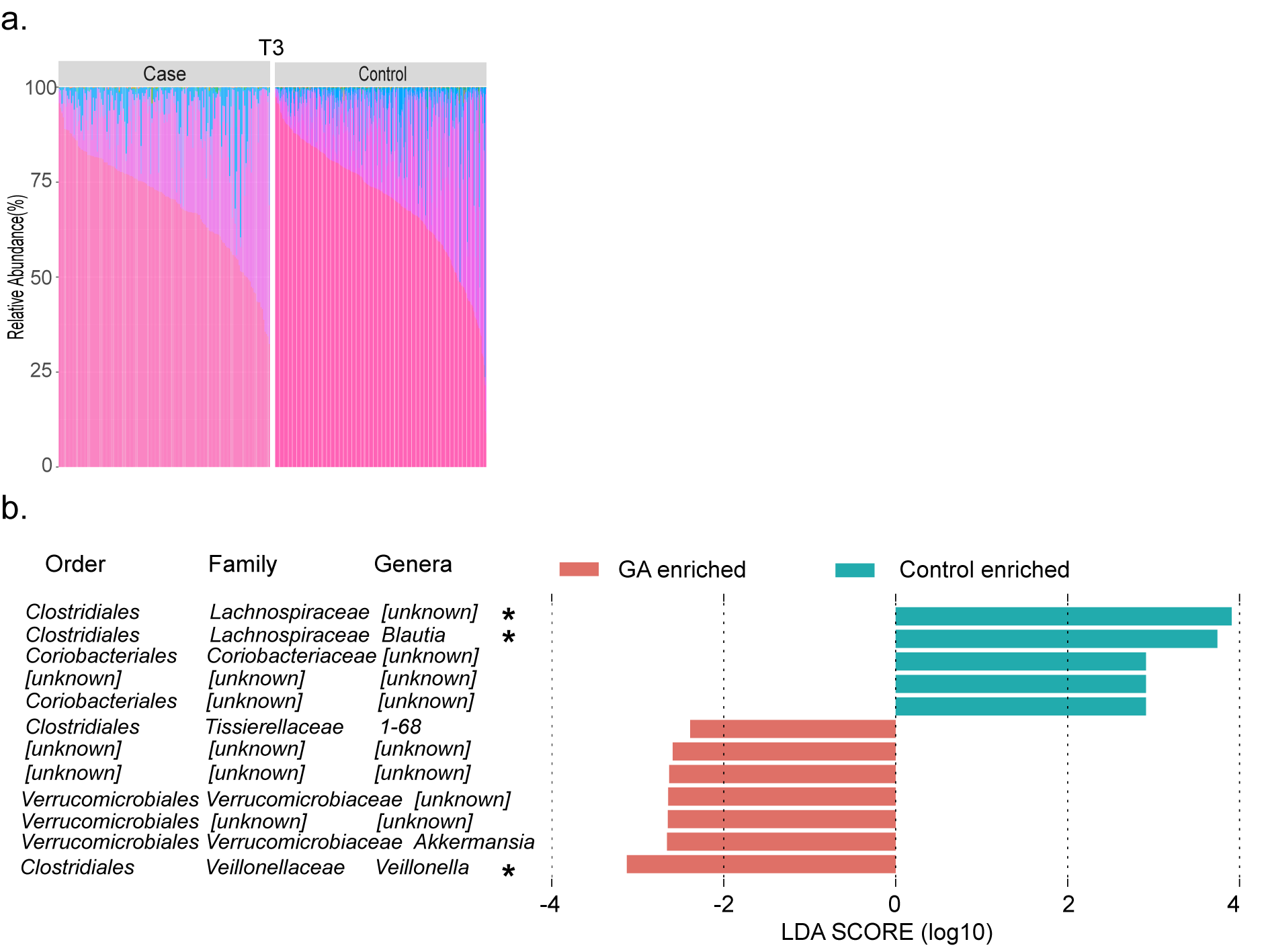

Supplement: Supplementary Figure 2 — (A) Relative proportions of bacterial phyla in GA and healthy control in the third trimester. (B) Histogram of the LDA scores computed for differentially abundant taxa between GA and healthy control in the third trimester. GA, gestational anemia; LDA, linear discriminant analysis; *Genera remained significantly associated with GA after adjusting for covariates using multivariate association with linear models algorithm (MaAsLin). [file Image_2.tif]
