# Supplementary material for: Insight Into the Potential Value of Gut Microbial Signatures for Prediction of Gestational Anemia
Source: Front Cell Infect Microbiol. 2021 Aug 30;11:734561. doi: 10.3389/fcimb.2021.734561 (PMC8437374; doi:10.3389/fcimb.2021.734561)
Supplement: Supplementary file 3 [file Table_1.docx]

Table S1. Comparisons of alpha-diversity and beta-diversity.

|  | *P*-value | | |
| --- | --- | --- | --- |
|  | observed_ASVs | PCoA based on weighted UniFrac matrix | |
| T2 vs. T3 |  |  |  |
| GA group | **<0.001** | **0.002** |  |
| Control group | **<0.001** | **0.001** |  |
| GA vs. Control |  |  |  |
| T2 | 0.63 | 0.86 |  |
| T3 | 0.20 | 0.21 |  |

Significant *P* valve (*P* < 0.05) is bold.

Table S2. Mean relative abundance of the different taxa at genera level between T2 and T3.

|  | Mean relative abundance (%) | |
| --- | --- | --- |
|  | T2 | T3 |
| k__Bacteria\|p__Bacteroidetes\|c__Bacteroidia | 15.3945 | 19.4252 |
| k__Bacteria\|p__Bacteroidetes\|c__Bacteroidia\|o__Bacteroidales | 15.3945 | 19.4252 |
| k__Bacteria\|p__Bacteroidetes | 0.0001 | 0.0003 |
| k__Bacteria\|p__Bacteroidetes\|c__Bacteroidia\|o__Bacteroidales\|f__Bacteroidaceae | 0.0000 | 0.0007 |
| k__Bacteria\|p__Bacteroidetes\|c__Bacteroidia\|o__Bacteroidales\|f__Bacteroidaceae\|g__Bacteroides | 8.0770 | 11.1044 |
| k__Bacteria\|p__Firmicutes\|c__Erysipelotrichi | 0.9511 | 1.3914 |
| k__Bacteria\|p__Firmicutes\|c__Erysipelotrichi\|o__Erysipelotrichales\|f__Erysipelotrichaceae\|__ | 0.0009 | 0.0017 |
| k__Bacteria\|p__Firmicutes\|c__Erysipelotrichi\|o__Erysipelotrichales | 0.9511 | 1.3914 |
| k__Bacteria\|p__Firmicutes\|c__Clostridia\|o__Clostridiales\|f__Lachnospiraceae\|g__ | 0.3412 | 0.6824 |
| k__Bacteria\|p__Firmicutes\|c__Clostridia\|o__Clostridiales\|f__Veillonellaceae\|g__Veillonella | 0.3256 | 0.5581 |
| k__Bacteria\|p__Firmicutes\|c__Erysipelotrichi\|o__Erysipelotrichales\|f__Erysipelotrichaceae\|g__ | 0.4126 | 0.6338 |
| k__Bacteria\|p__Firmicutes\|c__Clostridia\|o__Clostridiales\|f__Lachnospiraceae\|g__Clostridium | 0.3668 | 0.5528 |
| k__Bacteria\|p__Proteobacteria\|c__Betaproteobacteria\|o__Burkholderiales\|__\|__ | 0.0001 | 0.0003 |
| k__Bacteria\|p__Proteobacteria\|c__Betaproteobacteria\|__\|__\|__ | 0.0000 | 0.0000 |
| k__Bacteria\|p__Proteobacteria\|c__Betaproteobacteria\|o__Burkholderiales\|f__Alcaligenaceae\|g__Sutterella | 0.2372 | 0.4050 |
| k__Bacteria\|p__Proteobacteria\|c__Betaproteobacteria\|o__Burkholderiales\|f__Alcaligenaceae\|__ | 0.0000 | 0.0001 |
| k__Bacteria\|p__Bacteroidetes\|c__Bacteroidia\|o__Bacteroidales\|f__Porphyromonadaceae\|__ | 0.0000 | 0.0002 |
| k__Bacteria\|p__Bacteroidetes\|c__Bacteroidia\|o__Bacteroidales\|f__Porphyromonadaceae\|g__Parabacteroides | 0.3286 | 0.4715 |
| k__Bacteria\|p__Bacteroidetes\|c__Bacteroidia\|o__Bacteroidales\|f__Rikenellaceae\|g__Alistipes | 0.6374 | 0.7267 |
| k__Bacteria\|p__Bacteroidetes\|c__Bacteroidia\|o__Bacteroidales\|f__Rikenellaceae\|g__ | 0.2453 | 0.3321 |
| k__Bacteria\|p__Firmicutes\|c__Clostridia\|o__Clostridiales\|f__Peptostreptococcaceae | 0.0824 | 0.1599 |
| k__Bacteria\|p__Firmicutes\|c__Clostridia\|o__Clostridiales\|f__Lachnospiraceae\|g__Ruminococcus | 0.0022 | 0.0131 |
| k__Bacteria\|p__Bacteroidetes\|c__Bacteroidia\|o__Bacteroidales\|f__[Odoribacteraceae] | 0.1033 | 0.1805 |
| k__Bacteria\|p__Firmicutes\|c__Clostridia\|o__Clostridiales\|f__Peptostreptococcaceae\|__ | 0.0795 | 0.1369 |
| k__Bacteria\|p__Bacteroidetes\|c__Bacteroidia\|o__Bacteroidales\|f__[Odoribacteraceae]\|g__Odoribacter | 0.0581 | 0.0963 |
| k__Bacteria\|p__Bacteroidetes\|c__Bacteroidia\|o__Bacteroidales\|f__[Odoribacteraceae]\|g__Butyricimonas | 0.0452 | 0.0843 |
| k__Bacteria\|p__Verrucomicrobia\|c__Verrucomicrobiae\|o__Verrucomicrobiales | 0.0899 | 0.0947 |
| k__Bacteria\|p__Verrucomicrobia\|c__Verrucomicrobiae\|o__Verrucomicrobiales\|f__Verrucomicrobiaceae | 0.0899 | 0.0947 |
| k__Bacteria\|p__Verrucomicrobia\|c__Verrucomicrobiae | 0.0899 | 0.0947 |
| k__Bacteria\|p__Verrucomicrobia\|c__Verrucomicrobiae\|o__Verrucomicrobiales\|f__Verrucomicrobiaceae\|g__Akkermansia | 0.0899 | 0.0947 |
| k__Bacteria\|p__Verrucomicrobia | 0.0899 | 0.0947 |
|  |  |  |
| k__Bacteria\|p__Synergistetes\|c__Synergistia | 0.0514 | 0.0341 |
| k__Bacteria\|p__Tenericutes\|c__Mollicutes\|o__RF39 | 0.1390 | 0.0782 |
| k__Bacteria\|p__Tenericutes | 0.1397 | 0.0791 |
| k__Bacteria\|p__Tenericutes\|c__Mollicutes\|o__RF39\|f__ | 0.1390 | 0.0782 |
| k__Bacteria\|p__Tenericutes\|c__Mollicutes | 0.1391 | 0.0790 |
| k__Bacteria\|p__Tenericutes\|c__Mollicutes\|o__RF39\|f__\|g__ | 0.1390 | 0.0782 |
| k__Bacteria\|p__Proteobacteria\|c__Alphaproteobacteria\|o__Sphingomonadales\|__\|__ | 0.0004 | 0.0000 |
| k__Bacteria\|p__Firmicutes\|c__Clostridia\|o__Clostridiales\|f__Clostridiaceae | 0.9453 | 0.8628 |
| k__Bacteria\|p__Bacteroidetes\|c__Bacteroidia\|o__Bacteroidales\|f__Rikenellaceae\|__ | 0.2136 | 0.1360 |
| k__Bacteria\|p__Firmicutes\|c__Clostridia\|o__Clostridiales\|f__Clostridiaceae\|__ | 0.2861 | 0.1970 |
| k__Bacteria\|p__Actinobacteria\|c__Coriobacteriia\|o__Coriobacteriales\|f__Coriobacteriaceae\|g__ | 0.2182 | 0.1381 |
| k__Bacteria\|p__Chloroflexi\|c__Anaerolineae\|o__SBR1031\|f__A4b\|g__ | 0.0031 | 0.0000 |
| k__Bacteria\|p__Proteobacteria\|c__Alphaproteobacteria\|o__Sphingomonadales\|f__Sphingomonadaceae | 0.0076 | 0.0000 |
| k__Bacteria\|p__Proteobacteria\|c__Alphaproteobacteria\|o__Sphingomonadales\|f__Sphingomonadaceae\|__ | 0.0076 | 0.0000 |
| k__Bacteria\|p__Bacteroidetes\|c__[Saprospirae]\|o__[Saprospirales]\|f__Chitinophagaceae | 0.0013 | 0.0000 |
| k__Bacteria\|p__Proteobacteria\|c__Gammaproteobacteria\|o__Enterobacteriales\|f__Enterobacteriaceae\|g__Klebsiella | 0.4495 | 0.3617 |
| k__Bacteria\|p__Gemmatimonadetes\|c__Gemm-1\|o__ | 0.0054 | 0.0000 |
| k__Bacteria\|p__Chloroflexi\|c__Anaerolineae\|o__CFB-26\|f__\|g__ | 0.0004 | 0.0000 |
| k__Bacteria\|p__Gemmatimonadetes\|c__Gemm-1\|o__\|f__\|g__ | 0.0054 | 0.0000 |
| k__Bacteria\|p__Chloroflexi\|c__Anaerolineae\|o__CFB-26\|f__ | 0.0004 | 0.0000 |
| k__Bacteria\|p__Chloroflexi\|c__Anaerolineae\|o__SBR1031\|f__A4b | 0.0031 | 0.0000 |
| k__Bacteria\|p__Bacteroidetes\|c__[Saprospirae]\|o__[Saprospirales] | 0.0017 | 0.0000 |
| k__Bacteria\|p__Chloroflexi\|c__Anaerolineae\|o__CFB-26 | 0.0004 | 0.0000 |
| k__Bacteria\|p__Proteobacteria\|c__Betaproteobacteria\|o__SC-I-84 | 0.0011 | 0.0000 |
| k__Bacteria\|p__Gemmatimonadetes\|c__Gemm-1\|o__\|f__ | 0.0054 | 0.0000 |
| k__Bacteria\|p__Proteobacteria\|c__Betaproteobacteria\|o__SC-I-84\|f__ | 0.0011 | 0.0000 |
| k__Bacteria\|p__Chloroflexi\|c__Anaerolineae\|__\|__\|__ | 0.0001 | 0.0000 |
| k__Bacteria\|p__Gemmatimonadetes | 0.0077 | 0.0000 |
| k__Bacteria\|p__Gemmatimonadetes\|c__Gemmatimonadetes | 0.0077 | 0.0000 |
| k__Bacteria\|p__Proteobacteria\|c__Betaproteobacteria\|o__SC-I-84\|f__\|g__ | 0.0011 | 0.0000 |
| k__Bacteria\|p__Firmicutes\|c__Clostridia\|o__Clostridiales\|f__Lachnospiraceae\|g__Lachnobacterium | 0.3727 | 0.1876 |
| k__Bacteria\|p__Bacteroidetes\|c__Sphingobacteriia | 0.0004 | 0.0000 |
| k__Bacteria\|p__Chloroflexi\|c__Anaerolineae\|o__SBR1031 | 0.0031 | 0.0000 |
| k__Bacteria\|p__Chloroflexi | 0.0068 | 0.0000 |
| k__Bacteria\|p__Bacteroidetes\|c__[Saprospirae] | 0.0017 | 0.0000 |
| k__Bacteria\|p__Firmicutes\|c__Clostridia\|o__Clostridiales\|f__Christensenellaceae\|g__ | 0.8110 | 0.5976 |
| k__Bacteria\|p__Bacteroidetes\|c__Sphingobacteriia\|o__Sphingobacteriales\|f__Sphingobacteriaceae | 0.0002 | 0.0000 |
| k__Bacteria\|p__Gemmatimonadetes\|c__Gemm-1 | 0.0054 | 0.0000 |
| k__Bacteria\|p__Bacteroidetes\|c__Sphingobacteriia\|o__Sphingobacteriales | 0.0004 | 0.0000 |
| k__Bacteria\|p__Firmicutes\|c__Clostridia\|o__Clostridiales\|f__Christensenellaceae | 0.8241 | 0.6033 |
| k__Bacteria\|p__Proteobacteria\|c__Gammaproteobacteria\|o__Enterobacteriales\|f__Enterobacteriaceae\|__ | 3.0862 | 2.7583 |
| k__Bacteria\|p__Proteobacteria\|c__Gammaproteobacteria | 3.9693 | 3.6839 |
| k__Bacteria\|p__Firmicutes\|c__Clostridia\|o__Clostridiales\|__\|__ | 1.7638 | 1.3616 |
| k__Bacteria\|p__Firmicutes\|c__Clostridia\|o__Clostridiales\|__ | 1.7638 | 1.3616 |
| k__Bacteria\|p__Proteobacteria\|c__Gammaproteobacteria\|o__Enterobacteriales | 7.9658 | 3.8321 |
| k__Bacteria\|p__Proteobacteria\|c__Gammaproteobacteria\|o__Enterobacteriales\|f__Enterobacteriaceae | 3.8321 | 3.4707 |
| k__Bacteria\|p__Firmicutes\|c__Clostridia\|o__Clostridiales\|f__Ruminococcaceae\|g__Ruminococcus | 2.7242 | 2.2162 |
| k__Bacteria\|p__Firmicutes\|c__Clostridia\|o__Clostridiales\|f__Ruminococcaceae\|__ | 1.5893 | 1.0575 |
| k__Bacteria\|p__Firmicutes\|c__Clostridia\|o__Clostridiales\|f__ | 2.0463 | 1.4078 |
| k__Bacteria\|p__Firmicutes\|c__Clostridia\|o__Clostridiales\|f__\|g__ | 2.0463 | 1.4078 |
| k__Bacteria\|p__Firmicutes\|c__Clostridia\|o__Clostridiales\|f__Ruminococcaceae\|g__Oscillospira | 2.3004 | 1.7346 |
| k__Bacteria\|p__Firmicutes\|c__Clostridia\|o__Clostridiales\|f__Lachnospiraceae\|g__Pseudobutyrivibrio | 3.2181 | 2.7036 |
| k__Bacteria\|p__Firmicutes\|c__Clostridia\|o__Clostridiales\|f__Ruminococcaceae\|g__Gemmiger | 6.1676 | 5.4192 |
| k__Bacteria\|p__Firmicutes\|c__Clostridia\|o__Clostridiales\|f__Lachnospiraceae\|__ | 9.9722 | 8.6103 |
| k__Bacteria\|p__Firmicutes\|c__Clostridia\|o__Clostridiales\|f__Ruminococcaceae | 28.2184 | 25.2453 |
| k__Bacteria\|p__Firmicutes | 74.7638 | 70.2659 |
| k__Bacteria\|p__Firmicutes\|c__Clostridia | 72.0155 | 66.9198 |
| k__Bacteria\|p__Firmicutes\|c__Clostridia\|o__Clostridiales | 72.0155 | 66.9195 |

Table S3. Mean relative abundance of the different taxa at genera level between GA and healthy control.

|  | Mean relative abundance (%) | |
| --- | --- | --- |
|  | GA | Healthy control |
| k__Bacteria\|p__Firmicutes\|c__Clostridia\|o__Clostridiales\|f__Lachnospiraceae\|__ | 7.3370 | 9.0853 |
| k__Bacteria\|p__Firmicutes\|c__Clostridia\|o__Clostridiales\|f__Lachnospiraceae\|g__Blautia | 4.3776 | 5.4022 |
| k__Bacteria\|p__Actinobacteria\|c__Coriobacteriia\|o__Coriobacteriales\|f__Coriobacteriaceae | 1.0989 | 1.0761 |
|  |  |  |
| k__Bacteria\|p__Firmicutes\|c__Clostridia\|o__Clostridiales\|f__Veillonellaceae\|g__Megamonas | 2.5447 | 2.1205 |
| k__Bacteria\|p__Bacteroidetes\|c__Bacteroidia\|o__Bacteroidales\|f__[Paraprevotellaceae]\|__ | 0.0018 | 0.0000 |
| k__Bacteria\|p__Firmicutes\|c__Bacilli\|o__Gemellales | 0.0025 | 0.0003 |
| k__Bacteria\|p__Firmicutes\|c__Bacilli\|o__Gemellales\|f__Gemellaceae | 0.0025 | 0.0003 |
| k__Bacteria\|p__Fusobacteria\|c__Fusobacteriia\|o__Fusobacteriales\|f__Fusobacteriaceae\|g__Cetobacterium | 0.0628 | 0.0003 |
| k__Bacteria\|p__Proteobacteria\|c__Gammaproteobacteria\|o__Pasteurellales\|f__Pasteurellaceae\|g__Haemophilus | 0.1428 | 0.1241 |
| k__Bacteria\|p__Proteobacteria\|c__Gammaproteobacteria\|o__Pasteurellales | 0.1455 | 0.1258 |
| k__Bacteria\|p__Proteobacteria\|c__Gammaproteobacteria\|o__Pasteurellales\|f__Pasteurellaceae | 0.1455 | 0.1258 |
| k__Bacteria\|p__Proteobacteria\|c__Gammaproteobacteria\|o__Enterobacteriales\|f__Enterobacteriaceae\|g__Kluyvera | 0.0140 | 0.0005 |
| k__Bacteria\|p__Firmicutes\|c__Clostridia\|o__Clostridiales\|f__Veillonellaceae\|g__Veillonella | 0.7030 | 0.5040 |

Table S4. MaAsLin results of GA versus control in the second trimester.

| Taxa | Coefficient | | *P*-value |
| --- | --- | --- | --- |
| k__Bacteria\|p__Firmicutes\|c__Clostridia\|o__Clostridiales\|f__Veillonellaceae\|g__Veillonella | -0.01037 | 0.001994 | |
| k__Bacteria\|p__Firmicutes\|c__Clostridia\|o__Clostridiales\|f__Lachnospiraceae\|__ | 0.032296 | 0.008288 | |
| k__Bacteria\|p__Firmicutes\|c__Clostridia\|o__Clostridiales\|f__Lachnospiraceae\|g__Blautia | 0.022653 | 0.015525 | |
| k__Bacteria\|p__Proteobacteria\|c__Gammaproteobacteria\|o__Pasteurellales\|f__Pasteurellaceae\|g__Haemophilus | -0.00469 | 0.022305 | |
| k__Bacteria\|p__Firmicutes\|c__Clostridia\|o__Clostridiales\|f__Veillonellaceae\|g__Megamonas | -0.01426 | 0.023613 | |
| k__Bacteria\|p__Proteobacteria\|c__Gammaproteobacteria\|o__Enterobacteriales\|f__Enterobacteriaceae\|g__Klebsiella | -0.00932 | 0.058743 | |
| k__Bacteria\|p__Firmicutes\|c__Clostridia\|o__Clostridiales\|f__Clostridiaceae\|g__Clostridium | 0.006321 | 0.110553 | |
| k__Bacteria\|p__Bacteroidetes\|c__Bacteroidia\|o__Bacteroidales\|__\|__ | -0.00457 | 0.117563 | |
| k__Bacteria\|p__Proteobacteria\|c__Deltaproteobacteria\|o__Desulfovibrionales\|f__Desulfovibrionaceae\|g__Desulfovibrio | -0.00389 | 0.122552 | |
| k__Bacteria\|p__Firmicutes\|c__Erysipelotrichi\|o__Erysipelotrichales\|f__Erysipelotrichaceae\|g__Catenibacterium | 0.00469 | 0.174323 | |
| k__Bacteria\|p__Firmicutes\|c__Clostridia\|o__Clostridiales\|f__\|g__ | 0.00771 | 0.354247 | |
| k__Bacteria\|p__Firmicutes\|c__Clostridia\|o__Clostridiales\|f__Veillonellaceae\|__ | -0.00154 | 0.384227 | |
| k__Bacteria\|p__Proteobacteria\|c__Betaproteobacteria\|o__Burkholderiales\|f__Oxalobacteraceae\|g__Oxalobacter | -0.00032 | 0.392263 | |
| k__Bacteria\|p__Actinobacteria\|c__Coriobacteriia\|o__Coriobacteriales\|f__Coriobacteriaceae\|g__Collinsella | 0.003487 | 0.43477 | |
| k__Bacteria\|p__Firmicutes\|c__Clostridia\|o__Clostridiales\|f__Veillonellaceae\|g__Mitsuokella | -0.00185 | 0.454231 | |
| k__Bacteria\|p__Fusobacteria\|c__Fusobacteriia\|o__Fusobacteriales\|f__Fusobacteriaceae\|g__ | 0.000183 | 0.936824 | |

Significant *P* valve (*P* < 0.05) is bold.

Table S5. The top 99 most abundant genera were clustered into 8 CAGs in the second trimester.

| Taxa | CAG |
| --- | --- |
| k__Bacteria\|p__Firmicutes\|c__Clostridia\|o__Clostridiales\|f__Ruminococcaceae\|g__Faecalibacterium | CAG1 |
| k__Bacteria\|p__Bacteroidetes\|c__Bacteroidia\|o__Bacteroidales\|f__Bacteroidaceae\|g__Bacteroides |  |
| k__Bacteria\|p__Firmicutes\|c__Clostridia\|o__Clostridiales\|f__Lachnospiraceae\|g__Roseburia |  |
| k__Bacteria\|p__Firmicutes\|c__Clostridia\|o__Clostridiales\|f__Veillonellaceae\|g__Phascolarctobacterium |  |
| k__Bacteria\|p__Firmicutes\|c__Clostridia\|o__Clostridiales\|f__Lachnospiraceae\|g__Lachnospira |  |
| k__Bacteria\|p__Firmicutes\|c__Clostridia\|o__Clostridiales\|f__Lachnospiraceae\|g__Anaerostipes |  |
| k__Bacteria\|p__Proteobacteria\|c__Betaproteobacteria\|o__Burkholderiales\|f__Alcaligenaceae\|g__Sutterella |  |
| k__Bacteria\|p__Proteobacteria\|c__Deltaproteobacteria\|o__Desulfovibrionales\|f__Desulfovibrionaceae\|g__Bilophila |  |
| k__Bacteria\|p__Firmicutes\|c__Clostridia\|o__Clostridiales\|f__Lachnospiraceae\|__ | CAG2 |
| k__Bacteria\|p__Firmicutes\|c__Clostridia\|o__Clostridiales\|f__Lachnospiraceae\|g__Blautia |  |
| k__Bacteria\|p__Actinobacteria\|c__Actinobacteria\|o__Bifidobacteriales\|f__Bifidobacteriaceae\|g__Bifidobacterium |  |
| k__Bacteria\|p__Firmicutes\|c__Clostridia\|o__Clostridiales\|f__Clostridiaceae\|g__Clostridium |  |
| k__Bacteria\|p__Firmicutes\|c__Clostridia\|o__Clostridiales\|f__Ruminococcaceae\|g__Butyricicoccus |  |
| k__Bacteria\|p__Firmicutes\|c__Erysipelotrichi\|o__Erysipelotrichales\|f__Erysipelotrichaceae\|g__ |  |
| k__Bacteria\|p__Firmicutes\|c__Clostridia\|o__Clostridiales\|f__Lachnospiraceae\|g__Clostridium |  |
| k__Bacteria\|p__Firmicutes\|c__Clostridia\|o__Clostridiales\|f__Peptostreptococcaceae\|__ |  |
| k__Bacteria\|p__Firmicutes\|c__Erysipelotrichi\|o__Erysipelotrichales\|f__Erysipelotrichaceae\|g__Clostridium |  |
| k__Bacteria\|p__Actinobacteria\|c__Coriobacteriia\|o__Coriobacteriales\|f__Coriobacteriaceae\|g__Eggerthella |  |
| k__Bacteria\|p__Proteobacteria\|c__Gammaproteobacteria\|o__Enterobacteriales\|f__Enterobacteriaceae\|g__Escherichia |  |
| k__Bacteria\|p__Firmicutes\|c__Erysipelotrichi\|o__Erysipelotrichales\|f__Erysipelotrichaceae\|g__Holdemania |  |
| k__Bacteria\|p__Firmicutes\|c__Clostridia\|o__Clostridiales\|f__Ruminococcaceae\|g__Gemmiger | CAG3 |
| k__Bacteria\|p__Firmicutes\|c__Clostridia\|o__Clostridiales\|f__Lachnospiraceae\|g__Coprococcus |  |
| k__Bacteria\|p__Firmicutes\|c__Clostridia\|o__Clostridiales\|f__Ruminococcaceae\|g__Ruminococcus |  |
| k__Bacteria\|p__Firmicutes\|c__Clostridia\|o__Clostridiales\|f__Ruminococcaceae\|g__Oscillospira |  |
| k__Bacteria\|p__Firmicutes\|c__Clostridia\|o__Clostridiales\|f__\|g__ |  |
| k__Bacteria\|p__Firmicutes\|c__Clostridia\|o__Clostridiales\|__\|__ |  |
| k__Bacteria\|p__Firmicutes\|c__Clostridia\|o__Clostridiales\|f__Ruminococcaceae\|g__ |  |
| k__Bacteria\|p__Firmicutes\|c__Clostridia\|o__Clostridiales\|f__Ruminococcaceae\|__ |  |
| k__Bacteria\|p__Firmicutes\|c__Clostridia\|o__Clostridiales\|f__Christensenellaceae\|g__ |  |
| k__Bacteria\|p__Bacteroidetes\|c__Bacteroidia\|o__Bacteroidales\|f__Rikenellaceae\|g__Alistipes |  |
| k__Bacteria\|p__Firmicutes\|c__Clostridia\|o__Clostridiales\|f__Lachnospiraceae\|g__Lachnobacterium |  |
| k__Bacteria\|p__Bacteroidetes\|c__Bacteroidia\|o__Bacteroidales\|f__Porphyromonadaceae\|g__Parabacteroides |  |
| k__Bacteria\|p__Bacteroidetes\|c__Bacteroidia\|o__Bacteroidales\|f__Rikenellaceae\|g__ |  |
| k__Bacteria\|p__Actinobacteria\|c__Coriobacteriia\|o__Coriobacteriales\|f__Coriobacteriaceae\|g__ |  |
| k__Bacteria\|p__Bacteroidetes\|c__Bacteroidia\|o__Bacteroidales\|f__Rikenellaceae\|__ |  |
| k__Bacteria\|p__Bacteroidetes\|c__Bacteroidia\|o__Bacteroidales\|f__S24-7\|g__ |  |
| k__Bacteria\|p__Actinobacteria\|c__Coriobacteriia\|o__Coriobacteriales\|f__Coriobacteriaceae\|g__Adlercreutzia |  |
| k__Bacteria\|p__Tenericutes\|c__Mollicutes\|o__RF39\|f__\|g__ |  |
| k__Bacteria\|p__Bacteroidetes\|c__Bacteroidia\|o__Bacteroidales\|f__[Barnesiellaceae]\|g__ |  |
| k__Bacteria\|p__Bacteroidetes\|c__Bacteroidia\|o__Bacteroidales\|f__[Barnesiellaceae]\|__ |  |
| k__Bacteria\|p__Firmicutes\|c__Clostridia\|o__Clostridiales\|f__[Mogibacteriaceae]\|g__ |  |
| k__Bacteria\|p__Cyanobacteria\|c__4C0d-2\|o__YS2\|f__\|g__ |  |
| k__Bacteria\|p__Bacteroidetes\|c__Bacteroidia\|o__Bacteroidales\|f__[Odoribacteraceae]\|g__Odoribacter |  |
| k__Bacteria\|p__Bacteroidetes\|c__Bacteroidia\|o__Bacteroidales\|f__[Odoribacteraceae]\|g__Butyricimonas |  |
| k__Bacteria\|__\|__\|__\|__\|__ |  |
| k__Bacteria\|p__Synergistetes\|c__Synergistia\|o__Synergistales\|f__Synergistaceae\|g__Synergistes |  |
| k__Bacteria\|p__Actinobacteria\|c__Coriobacteriia\|o__Coriobacteriales\|f__Coriobacteriaceae\|g__Slackia |  |
| k__Bacteria\|p__Firmicutes\|c__Clostridia\|o__Clostridiales\|f__Ruminococcaceae\|g__Clostridium |  |
| k__Bacteria\|p__Bacteroidetes\|c__Bacteroidia\|o__Bacteroidales\|f__Prevotellaceae\|g__Prevotella | CAG4 |
| k__Bacteria\|p__Firmicutes\|c__Clostridia\|o__Clostridiales\|f__Veillonellaceae\|g__Dialister |  |
| k__Bacteria\|p__Firmicutes\|c__Clostridia\|o__Clostridiales\|f__Veillonellaceae\|g__Megamonas |  |
| k__Bacteria\|p__Bacteroidetes\|c__Bacteroidia\|o__Bacteroidales\|f__[Paraprevotellaceae]\|g__Paraprevotella |  |
| k__Bacteria\|p__Bacteroidetes\|c__Bacteroidia\|o__Bacteroidales\|__\|__ |  |
| k__Bacteria\|p__Firmicutes\|c__Erysipelotrichi\|o__Erysipelotrichales\|f__Erysipelotrichaceae\|g__Catenibacterium |  |
| k__Bacteria\|p__Proteobacteria\|c__Deltaproteobacteria\|o__Desulfovibrionales\|f__Desulfovibrionaceae\|g__Desulfovibrio |  |
| k__Bacteria\|p__Bacteroidetes\|c__Bacteroidia\|o__Bacteroidales\|f__[Paraprevotellaceae]\|g__[Prevotella] |  |
| k__Bacteria\|p__Firmicutes\|c__Clostridia\|o__Clostridiales\|f__Veillonellaceae\|g__Mitsuokella |  |
| k__Bacteria\|p__Firmicutes\|c__Clostridia\|o__Clostridiales\|f__Veillonellaceae\|__ |  |
| k__Bacteria\|p__Firmicutes\|c__Clostridia\|o__Clostridiales\|f__Peptococcaceae\|g__Peptococcus |  |
| k__Bacteria\|p__Bacteroidetes\|c__Bacteroidia\|o__Bacteroidales\|f__[Paraprevotellaceae]\|g__ |  |
| k__Bacteria\|p__Firmicutes\|c__Clostridia\|o__Clostridiales\|f__Lachnospiraceae\|g__Butyrivibrio |  |
| k__Bacteria\|p__Firmicutes\|c__Clostridia\|o__Clostridiales\|f__Lachnospiraceae\|g__Pseudobutyrivibrio | CAG5 |
| k__Bacteria\|p__Firmicutes\|c__Clostridia\|o__Clostridiales\|f__Veillonellaceae\|g__Megasphaera |  |
| k__Bacteria\|p__Fusobacteria\|c__Fusobacteriia\|o__Fusobacteriales\|f__Fusobacteriaceae\|g__Cetobacterium |  |
| k__Bacteria\|p__Firmicutes\|c__Clostridia\|o__Clostridiales\|f__Veillonellaceae\|g__Acidaminococcus |  |
| k__Bacteria\|p__Bacteroidetes\|c__Bacteroidia\|o__Bacteroidales\|f__Rikenellaceae\|g__human |  |
| k__Bacteria\|p__Proteobacteria\|c__Gammaproteobacteria\|o__Enterobacteriales\|f__Enterobacteriaceae\|__ | CAG6 |
| k__Bacteria\|p__Firmicutes\|c__Bacilli\|o__Lactobacillales\|f__Streptococcaceae\|g__Streptococcus |  |
| k__Bacteria\|p__Firmicutes\|c__Clostridia\|o__Clostridiales\|f__Lachnospiraceae\|g__[Ruminococcus] |  |
| k__Bacteria\|p__Proteobacteria\|c__Gammaproteobacteria\|o__Enterobacteriales\|f__Enterobacteriaceae\|g__Klebsiella |  |
| k__Bacteria\|p__Firmicutes\|c__Clostridia\|o__Clostridiales\|f__Lachnospiraceae\|g__ |  |
| k__Bacteria\|p__Firmicutes\|c__Clostridia\|o__Clostridiales\|f__Veillonellaceae\|g__Veillonella |  |
| k__Bacteria\|p__Proteobacteria\|c__Gammaproteobacteria\|o__Enterobacteriales\|f__Enterobacteriaceae\|g__Enterobacter |  |
| k__Bacteria\|p__Firmicutes\|c__Bacilli\|o__Lactobacillales\|f__Lactobacillaceae\|g__Lactobacillus |  |
| k__Bacteria\|p__Proteobacteria\|c__Gammaproteobacteria\|o__Pasteurellales\|f__Pasteurellaceae\|g__Haemophilus |  |
| k__Bacteria\|p__Fusobacteria\|c__Fusobacteriia\|o__Fusobacteriales\|f__Fusobacteriaceae\|g__ |  |
| k__Bacteria\|p__Proteobacteria\|c__Gammaproteobacteria\|o__Enterobacteriales\|f__Enterobacteriaceae\|g__Citrobacter |  |
| k__Bacteria\|p__TM7\|c__TM7-3\|o__\|f__\|g__ |  |
| k__Bacteria\|p__Actinobacteria\|c__Actinobacteria\|o__Actinomycetales\|f__Micrococcaceae\|g__Rothia |  |
| k__Bacteria\|p__Fusobacteria\|c__Fusobacteriia\|o__Fusobacteriales\|f__Fusobacteriaceae\|__ |  |
| k__Bacteria\|p__Proteobacteria\|c__Alphaproteobacteria\|o__Rhizobiales\|f__Bradyrhizobiaceae\|__ |  |
| k__Bacteria\|p__Firmicutes\|c__Clostridia\|o__Clostridiales\|f__Lachnospiraceae\|g__Dorea | CAG7 |
| k__Bacteria\|p__Actinobacteria\|c__Coriobacteriia\|o__Coriobacteriales\|f__Coriobacteriaceae\|g__Collinsella |  |
| k__Bacteria\|p__Firmicutes\|c__Erysipelotrichi\|o__Erysipelotrichales\|f__Erysipelotrichaceae\|g__[Eubacterium] |  |
| k__Bacteria\|p__Firmicutes\|c__Clostridia\|o__Clostridiales\|f__Clostridiaceae\|__ |  |
| k__Bacteria\|p__Firmicutes\|c__Bacilli\|o__Turicibacterales\|f__Turicibacteraceae\|g__Turicibacter |  |
| k__Bacteria\|p__Firmicutes\|c__Bacilli\|o__Lactobacillales\|f__Streptococcaceae\|g__Lactococcus |  |
| k__Bacteria\|p__Firmicutes\|c__Bacilli\|o__Lactobacillales\|f__Enterococcaceae\|g__Enterococcus |  |
| k__Bacteria\|p__Cyanobacteria\|c__Chloroplast\|o__Streptophyta\|f__\|g__ |  |
| k__Bacteria\|p__Firmicutes\|c__Bacilli\|o__Lactobacillales\|f__Leuconostocaceae\|g__Weissella |  |
| k__Bacteria\|p__Actinobacteria\|c__Actinobacteria\|o__Actinomycetales\|f__Actinosynnemataceae\|__ |  |
| k__Bacteria\|p__Proteobacteria\|c__Alphaproteobacteria\|o__Sphingomonadales\|f__Sphingomonadaceae\|__ |  |
| k__Bacteria\|p__Actinobacteria\|c__Coriobacteriia\|o__Coriobacteriales\|f__Coriobacteriaceae\|__ | CAG8 |
| k__Bacteria\|p__Verrucomicrobia\|c__Verrucomicrobiae\|o__Verrucomicrobiales\|f__Verrucomicrobiaceae\|g__Akkermansia |  |
| k__Bacteria\|p__Bacteroidetes\|c__Bacteroidia\|o__Bacteroidales\|f__[Barnesiellaceae]\|g__Barnesiella |  |
| k__Bacteria\|p__Synergistetes\|c__Synergistia\|o__Synergistales\|f__Synergistaceae\|g__Cloacibacillus |  |
| k__Bacteria\|p__Firmicutes\|c__Clostridia\|o__Clostridiales\|f__Christensenellaceae\|g__Christensenella |  |
| k__Bacteria\|p__Firmicutes\|c__Clostridia\|o__Clostridiales\|f__Ruminococcaceae\|g__Anaerotruncus |  |
| k__Bacteria\|p__Firmicutes\|c__Clostridia\|o__Clostridiales\|f__Clostridiaceae\|g__ |  |

Table S6. Associations between gestational anemia and clinical indices in the second trimester.

|  | Model 1 OR (95%CI) | *P* | Model 2 OR (95%CI) | *P* |
| --- | --- | --- | --- | --- |
| Blood urea nitrogen (mmol/L) | 1.05 (0.78, 1.41) | 0.75 | 1.65 (0.90, 3.02) | 0.10 |
| Creatinine (μmol/L) | 0.98 (0.95, 1.01) | 0.20 | 1.03 (0.96, 1.10) | 0.40 |
| Uric acid (μmol/L) | 0.999 (0.995, 1.003) | 0.73 | 1.00 (0.997, 1.01) | 0.26 |
| Total protein (g/L) | 0.999 (0.94, 1.06) | 0.98 | 1.03 (0.90, 1.17) | 0.67 |
| Albumin (g/L) | 0.90 (0.82, 0.99) | 0.03 | 0.73 (0.57, 0.93) | 0.01 |
| Cholesterol (mmol/L) | 0.99 (0.80, 1.21) | 0.90 | 1.20 (0.79, 1.84) | 0.40 |
| Triglyceride (mmol/L) | 1.03 (0.80, 1.33) | 0.82 | 1.41 (0.83, 2.40) | 0.20 |
| Alanine transaminase (U/L) | 0.99 (0.97, 1.01) | 0.22 | 0.99 (0.96, 1.03) | 0.67 |
| Aspartate transaminase (U/L) | 0.99 (0.96, 1.02) | 0.38 | 0.96 (0.89, 1.02) | 0.20 |
| Alkaline phosphatase (U/L) | 1.01 (0.99, 1.03) | 0.30 | 1.04 (1.00, 1.08) | 0.07 |
| Lactate dehydrogenase (U/L) | 1.00 (0.99, 1.01) | 0.94 | 1.00 (0.99, 1.02) | 0.58 |
| γ-glutamyl transpeptidase (U/L) | 0.97 (0.95, 1.00) | 0.049 | 0.99 (0.94, 1.04) | 0.58 |
| Total bilirubin (μmol/L) | 0.84 (0.76, 0.93) | 0.001 | 0.79 (0.65, 0.95) | 0.02 |
| Direct bilirubin (μmol/L) | 0.63 (0.47, 0.84) | 0.001 | 0.50 (0.28, 0.87) | 0.02 |
| Total bile acid (μmol/L) | 1.04 (0.89, 1.23) | 0.61 | 1.08 (0.71, 1.65) | 0.71 |
| Fructosamine (mmol/L) | 1.46 (0.92, 2.30) | 0.11 | 4.42 (0.85, 23.03) | 0.08 |
| Glucose (mmol/L) | 1.02 (0.53, 1.98) | 0.94 | 0.97 (0.23, 4.10) | 0.96 |
| Free thyroxine (pmol/L) | 0.82 (0.72, 0.93) | <0.01 | 0.73 (0.55, 0.96) | 0.03 |
| White blood cell (×10^9^/L) | 1.00 (0.92, 1.09) | 0.92 | 1.12 (0.94, 1.33) | 0.21 |
| Red blood cell (×10^12^/L) | 0.08 (0.04, 0.18) | <0.001 | 0.05 (0.01, 0.29) | <0.01 |
| Hemoglobin (g/L) | 0.85 (0.82, 0.89) | <0.001 | 0.84 (0.77, 0.90) | <0.001 |
| Blood platelet (×10^9^/L) | 1.001 (1.00, 1.01) | 0.48 | 1.00 (0.99, 1.01) | 0.66 |

Statistically significant results (*P* < 0.05) are bolded.

Model 1 was crude model; Model 2 was adjusted for maternal age, pre pregnant BMI (body mass index), parity, gravidity, family income, maternal education level, passive smoking, antibiotic use during pregnancy, folic acid and iron supplement use during pregnancy, alcohol and caffeine use pre pregnancy, alcohol and caffeine use during pregnancy.

Table S7. Association between gestational anemia and serum ferritin in the second trimester.

|  | Model 1 OR (95%CI) | *P* | Model 2 OR (95%CI) | *P* |
| --- | --- | --- | --- | --- |
| Serum ferritin | 1.00 (1.00, 1.00) | 0.39 | 1.00 (0.999, 1.00) | 0.46 |

Model 1 was crude model; Model 2 was adjusted for maternal age, pre pregnant BMI (body mass index), parity, gravidity, family income, maternal education level, passive smoking, antibiotic use during pregnancy, folic acid and iron supplement use during pregnancy, alcohol and caffeine use pre pregnancy, alcohol and caffeine use during pregnancy.

Table S8. Partial Spearman correlation coefficients between 13 taxa and clinical indices in the second trimester.

|  | *Megamonas* | *Paraprevotellaceae* | *Gemellales* | *Gemellaceae* | *Cetobacterium* | *Haemophilus* | *Pasteurellales* | *Pasteurellaceae* | *Kluyvera* | *Veillonella* | *Lachnospiraceae* | *Blautia* | *Coriobacteriaceae* |
| --- | --- | --- | --- | --- | --- | --- | --- | --- | --- | --- | --- | --- | --- |
| BUN | -0.033 | -0.049 | 0.016 | 0.034 | 0.098 | 0.01 | -0.028 | 0.023 | 0.052 | 0.042 | -0.021 | -0.074 | -0.056 |
| Cr | -0.09 | 0.011 | -0.005 | 0.029 | -0.027 | 0.081 | -0.045 | 0.041 | 0.013 | -0.021 | 0.04 | -0.058 | -0.056 |
| UA | 0.011 | -0.022 | 0.03 | -0.008 | 0.074 | 0.04 | -0.048 | -0.011 | 0.005 | 0.063 | 0.126 | 0.075 | -0.008 |
| TP | -0.011 | 0.01 | 0.035 | 0.071 | 0.094 | -0.05 | 0.007 | 0.018 | 0.019 | 0.029 | 0.064 | 0.059 | -0.04 |
| ALB | -0.07 | 0.004 | -0.016 | 0.038 | 0.045 | -0.077 | -0.023 | 0.018 | 0.024 | -0.014 | 0.12 | 0.04 | -0.011 |
| CHOL | 0.044 | 0.022 | 0.11 | -0.034 | 0.083 | 0.026 | -0.029 | 0.05 | 0.001 | -0.02 | -0.002 | 0.107 | 0.021 |
| TG | 0.03 | -0.029 | 0.086 | 0.064 | 0.036 | 0 | -0.071 | -0.029 | 0.002 | -0.028 | 0.038 | 0.167 | -0.027 |
| ALT | -0.026 | -0.042 | -0.008 | -0.052 | -0.066 | 0.183 | 0.053 | -0.018 | 0.026 | 0.02 | 0.088 | 0.05 | -0.038 |
| AST | 0.036 | 0.011 | 0.01 | -0.042 | -0.06 | 0.195 | 0.061 | 0.036 | -0.003 | 0.004 | 0.042 | 0.009 | -0.04 |
| ALP | 0.088 | -0.076 | 0.049 | 0.024 | -0.025 | 0.032 | 0.035 | 0.014 | 0.009 | 0.096 | -0.01 | 0.047 | -0.147 |
| LDH | 0.017 | -0.032 | 0.023 | -0.052 | -0.002 | 0.094 | 0.073 | -0.029 | 0.07 | 0.095 | -0.035 | -0.064 | -0.064 |
| GGT | 0.001 | -0.02 | 0.038 | -0.057 | -0.111 | 0.051 | 0.014 | -0.011 | -0.026 | -0.069 | 0.057 | 0.084 | -0.064 |
| TB | -0.023 | 0.034 | 0.034 | -0.053 | 0.018 | 0.02 | 0 | 0.083 | 0.077 | 0.016 | 0.004 | -0.099 | -0.136 |
| DB | -0.026 | 0.014 | 0.037 | -0.066 | -0.029 | -0.023 | -0.001 | 0.028 | 0.066 | -0.038 | -0.001 | -0.139 | -0.099 |
| TBA | 0.014 | -0.07 | -0.001 | -0.015 | -0.032 | 0.015 | -0.068 | 0.025 | -0.014 | -0.048 | 0.014 | -0.071 | 0 |
| FMN | 0.021 | 0.035 | -0.038 | 0.067 | 0.002 | 0.015 | -0.02 | 0.026 | 0.038 | 0.047 | 0.02 | -0.032 | -0.052 |
| GLU | 0.019 | 0.002 | 0 | 0.023 | -0.051 | -0.051 | 0.015 | -0.056 | 0.02 | -0.088 | 0.018 | -0.06 | -0.058 |
| FT4 | -0.109 | 0.106 | -0.121 | -0.011 | 0.016 | -0.009 | -0.027 | 0.017 | 0.07 | 0.052 | 0.055 | 0.014 | -0.037 |
| WBC | -0.02 | -0.1 | -0.007 | -0.002 | -0.039 | 0.018 | 0.042 | -0.098 | 0.037 | 0.012 | 0 | 0.063 | -0.02 |
| RBC | 0.077 | -0.001 | -0.036 | -0.065 | 0.037 | -0.053 | 0.007 | -0.05 | -0.01 | 0.001 | -0.024 | -0.008 | -0.039 |
| Hb | 0.028 | 0.063 | -0.056 | -0.059 | 0.031 | 0.005 | 0.013 | -0.024 | -0.003 | 0.009 | 0.013 | -0.027 | -0.039 |
| PLT | -0.058 | -0.048 | 0.037 | 0.061 | 0.03 | -0.009 | -0.042 | -0.019 | -0.015 | 0.004 | -0.041 | 0.006 | -0.020 |

Table S9. P values of partial Spearman correlation coefficients between 13 taxa and clinical indices in the second trimester.

|  | *Megamonas* | *Paraprevotellaceae* | *Gemellales* | *Gemellaceae* | *Cetobacterium* | *Haemophilus* | *Pasteurellales* | *Pasteurellaceae* | *Kluyvera* | *Veillonella* | *Lachnospiraceae* | *Blautia* | *Coriobacteriaceae* |
| --- | --- | --- | --- | --- | --- | --- | --- | --- | --- | --- | --- | --- | --- |
| BUN | 0.433 | 0.246 | 0.712 | 0.425 | 0.02 | 0.822 | 0.516 | 0.595 | 0.217 | 0.311 | 0.598 | 0.325 | 0.241 |
| Cr | 0.034 | 0.803 | 0.903 | 0.49 | 0.52 | 0.057 | 0.284 | 0.336 | 0.767 | 0.627 | 0.35 | 0.176 | 0.191 |
| UA | 0.794 | 0.606 | 0.479 | 0.859 | 0.083 | 0.343 | 0.255 | 0.793 | 0.902 | 0.137 | 0.003 | 0.077 | 0.849 |
| TP | 0.798 | 0.822 | 0.41 | 0.093 | 0.027 | 0.243 | 0.874 | 0.675 | 0.655 | 0.501 | 0.135 | 0.168 | 0.347 |
| ALB | 0.097 | 0.925 | 0.709 | 0.371 | 0.288 | 0.068 | 0.587 | 0.678 | 0.57 | 0.746 | 0.005 | 0.352 | 0.803 |
| CHOL | 0.302 | 0.604 | 0.009 | 0.423 | 0.049 | 0.539 | 0.492 | 0.234 | 0.973 | 0.632 | 0.954 | 0.012 | 0.622 |
| TG | 0.473 | 0.49 | 0.042 | 0.128 | 0.397 | 0.994 | 0.093 | 0.491 | 0.969 | 0.516 | 0.376 | 0.000 | 0.527 |
| ALT | 0.545 | 0.318 | 0.842 | 0.222 | 0.122 | 0.000 | 0.209 | 0.667 | 0.535 | 0.632 | 0.039 | 0.241 | 0.375 |
| AST | 0.397 | 0.792 | 0.815 | 0.326 | 0.157 | 0.000 | 0.153 | 0.397 | 0.937 | 0.929 | 0.318 | 0.838 | 0.348 |
| ALP | 0.038 | 0.075 | 0.251 | 0.575 | 0.554 | 0.445 | 0.408 | 0.741 | 0.834 | 0.024 | 0.812 | 0.264 | 0.000 |
| LDH | 0.695 | 0.448 | 0.59 | 0.22 | 0.971 | 0.027 | 0.087 | 0.497 | 0.099 | 0.025 | 0.411 | 0.132 | 0.134 |
| GGT | 0.988 | 0.635 | 0.368 | 0.178 | 0.009 | 0.234 | 0.744 | 0.803 | 0.546 | 0.107 | 0.178 | 0.049 | 0.134 |
| TB | 0.59 | 0.429 | 0.427 | 0.21 | 0.671 | 0.63 | 0.995 | 0.051 | 0.071 | 0.705 | 0.925 | 0.02 | 0.001 |
| DB | 0.537 | 0.745 | 0.379 | 0.118 | 0.499 | 0.582 | 0.973 | 0.503 | 0.12 | 0.366 | 0.974 | 0.001 | 0.020 |
| TBA | 0.74 | 0.1 | 0.976 | 0.732 | 0.447 | 0.721 | 0.111 | 0.548 | 0.738 | 0.255 | 0.734 | 0.096 | 0.996 |
| FMN | 0.621 | 0.414 | 0.366 | 0.116 | 0.966 | 0.716 | 0.641 | 0.538 | 0.366 | 0.269 | 0.636 | 0.450 | 0.217 |
| GLU | 0.662 | 0.956 | 0.995 | 0.58 | 0.226 | 0.228 | 0.728 | 0.188 | 0.638 | 0.039 | 0.667 | 0.158 | 0.175 |
| FT4 | 0.01 | 0.013 | 0.004 | 0.789 | 0.714 | 0.827 | 0.526 | 0.69 | 0.098 | 0.219 | 0.196 | 0.736 | 0.384 |
| WBC | 0.633 | 0.019 | 0.862 | 0.96 | 0.352 | 0.667 | 0.325 | 0.021 | 0.381 | 0.771 | 0.994 | 0.136 | 0.632 |
| RBC | 0.071 | 0.986 | 0.396 | 0.125 | 0.378 | 0.208 | 0.872 | 0.236 | 0.814 | 0.981 | 0.573 | 0.859 | 0.362 |
| Hb | 0.505 | 0.138 | 0.188 | 0.164 | 0.465 | 0.913 | 0.756 | 0.576 | 0.936 | 0.836 | 0.756 | 0.52 | 0.357 |
| PLT | 0.174 | 0.261 | 0.379 | 0.149 | 0.473 | 0.837 | 0.323 | 0.658 | 0.723 | 0.92 | 0.331 | 0.889 | 0.642 |

Statistically significant results (*P* < 0.05) are bold.
